# Hydration level is an internal variable for computing motivation to obtain water rewards in monkeys

**DOI:** 10.1007/s00221-012-3054-3

**Published:** 2012-03-13

**Authors:** Takafumi Minamimoto, Hiroshi Yamada, Yukiko Hori, Tetsuya Suhara

**Affiliations:** 1 Department of Molecular Neuroimaging, Molecular Imaging Center, National Institute of Radiological Sciences, Chiba, 263-8555 Japan; 2 PRESTO, Japan Science and Technology Agency (JST), 4-1-8 Honcho Kawaguchi, Saitama, 332-0012 Japan; 3 Center for Neural Science, New York University, New York, NY 10003 USA; 4 National Center of Neurology and Psychiatry 4-1-1 Ogawa-Higashi, Kodaira, Tokyo, 187-8502 Japan

**Keywords:** Motivation, Osmolality, Satiation, Thirst, Drive

## Abstract

In the process of motivation to engage in a behavior, valuation of the expected outcome is comprised of not only external variables (i.e., incentives) but also internal variables (i.e., drive). However, the exact neural mechanism that integrates these variables for the computation of motivational value remains unclear. Besides, the signal of physiological needs, which serves as the primary internal variable for this computation, remains to be identified. Concerning fluid rewards, the osmolality level, one of the physiological indices for the level of thirst, may be an internal variable for valuation, since an increase in the osmolality level induces drinking behavior. Here, to examine the relationship between osmolality and the motivational value of a water reward, we repeatedly measured the blood osmolality level, while 2 monkeys continuously performed an instrumental task until they spontaneously stopped. We found that, as the total amount of water earned increased, the osmolality level progressively decreased (i.e., the hydration level increased) in an individual-dependent manner. There was a significant negative correlation between the error rate of the task (the proportion of trials with low motivation) and the osmolality level. We also found that the increase in the error rate with reward accumulation can be well explained by a formula describing the changes in the osmolality level. These results provide a biologically supported computational formula for the motivational value of a water reward that depends on the hydration level, enabling us to identify the neural mechanism that integrates internal and external variables.

## Introduction

The valuation of expected outcomes is one of the critical processes underlying value-based decision-making and the motivation to engage in a behavior. In the motivational process, the valuation of the outcome must be subjective; it must take into account not only external variables (e.g., size and type of rewards) but also internal variables (e.g., hunger or thirst). Indeed, changes in the internal state of the physiological need (e.g., from hunger to satiation) for specific rewards affect the behavior to attain these rewards (Dickinson and Balleine 1994). For example, instrumental behavior that leads to a food reward is suppressed after subjects are satiated, suggesting that the value of the food is discounted (known as reinforcer devaluation) (Baxter and Murray 2002). Accumulating evidence has suggested that the neural basis of reinforcer devaluation is distributed across the orbitofrontal cortex, amygdala, and mediodorsal nucleus of the thalamus (Izquierdo and Murray 2010; Izquierdo et al. 2004; Machado and Bachevalier 2007; Malkova et al. 1997; Rudebeck et al. 2008). It is also known that the activity of the brain regions that represent the value of rewards depends on the subject’s internal state (e.g., sated or not) (Bouret and Richmond 2010; Critchley and Rolls 1996; de Araujo et al. 2003; Simon et al. 2006). However, the exact neural mechanism by which internal and external variables are integrated to compute the motivational value of outcome remains unclear. Specifically, the following remain to be identified: (1) the signal of physiological needs that serves as the primary internal variable for computing the motivational value and (2) the exact form of this computation, that is, the integration of internal and external variables.

During behavioral testing, the external variables (e.g., reward size) can be easily manipulated on a trial-by-trial basis, whereas the internal variables (e.g., satiation level) cannot be controlled precisely. Accordingly, one can solve these issues by assessing the reward valuation in a behavioral task while the level of physiological needs is monitored. The level of need for water (i.e., hydration state) can be inferred by measuring blood osmolality (Yamada et al. 2010), which is the most widely used hematological index of hydration status. It is widely known that mammals control their body fluid balance by maintaining their osmolality level at a common “set-point” (~300 mOsm/kgH_2_O) (Bourque 2008). The sensation of thirst and spontaneous drinking are elicited by increases in the blood osmolality level induced by the intravenous infusion of hypertonic saline (Anderson and Houpt 1990; Egan et al. 2003). The drinking induced by blood hyperosmolality can be terminated when normal osmolality is restored (Houpt et al. 1999). These observations suggest that the need for water (and value of water) is increased by a rise in osmolality above the normal level, whereas it is decreased at the normal osmolality level. Therefore, the osmotic signal can be utilized as one of the internal variables for the valuation of water rewards.

**Fig. 1:**
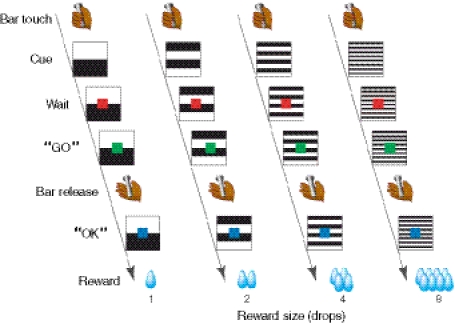
Behavioral paradigm. Sequence of events during a trial of the reward-size task. A monkey initiated a trial by touching the bar in the chair. The visual cue presented at the beginning of the trial (“Cue”; *black* and *white* stripes) indicated the number of drops for the reward. The monkey was required to release the bar to earn a liquid reward after the red signal (“Wait”) turned to green (“GO”). In the correct trials, the assigned number of drops was delivered immediately after the blue signal (“OK”) and then followed by the next trial in which the reward size was selected randomly with equal probability. If the monkey released the bar before GO or within 200 ms after GO or failed to respond within 1 s after GO, we regarded the trial as an “error trial.” The error trial was repeated with the same reward size

To investigate how motivational value is derived from internal and external variables, behavioral tasks have been designed with water reward outcomes for macaque monkeys (Minamimoto et al. 2009). In one of these tasks, named the “reward-size task” (Fig. 1), the monkeys are required to release a bar after a red light turns green to receive a water reward. The amount of reward varies trial by trial, and it is indicated by a visual cue at the beginning of each trial. The monkeys are gradually rehydrated over the course of the task, which is usually a few hours, by sipping the water rewards obtained in every successful trial, as performed in typical behavioral tasks used in electrophysiological studies of monkeys (e.g., Minamimoto et al. 2005). The error rate, that is, the proportion of trials in which the monkeys did not engage in this instrumental action, is used as a measure of the motivational value; the error rate is well described by a model in which the expected reward amount (i.e., external variable) is multiplied by a decay function according to water consumption (i.e., inference of internal variable) (Minamimoto et al. 2009).

In the present study, we examined the relationship between the osmotic signal and the motivational value of water rewards. We repeatedly measured the blood osmolality level while 2 monkeys continuously performed a reward-size task for consuming water rewards, slowly moving from thirst to satiation until they spontaneously quit the task. We further sought to identify a new model explaining the increases in the error rate along with reward accumulation by the decrease in the osmolality level. The identification of this model could allow us to seek the neural basis for the computation of motivational value with individual internal variables.

## Materials and methods

### Subjects

The subjects were 2 male rhesus monkeys (*monkeys LP and GM,* 7.8 and 5.6 kg, respectively). Body weight was measured once every 2 weeks on average throughout the experimental period. All experimental procedures were carried out in accordance with the National Institutes of Health Guide for the Care and Use of Laboratory Animals in the USA and were approved by the Animal Care and Use Committee of the National Institute of Radiological Sciences.

### Measurement of blood osmolality

Blood samples (~0.5 mL/sample) were drawn from the saphenous vein via a venous catheter using an auto-blood sampling system (DR-II, Eicom Co.; in 7 sessions) or manually (in 3 sessions). The samples were stored at 4 °C for up to 3 h. After blood collection, blood osmolality was measured by using a freezing point method (Advance 3250, Advanced Instruments Inc.) on whole blood samples of 250 μL. The measurement error was ~2 mOsm/kgH_2_O. Since there was no significant difference in the osmolality level between serum and whole blood samples (0 ± 2.7 mOsm/kgH_2_O; difference ± SD; *n* = 9), we used whole blood samples to reduce the total sampling volume. The total amount of blood sampled never exceeded 4 mL in a day.

### Behavioral task and testing procedure

We used the reward-size task (Fig. 1) (Minamimoto et al. 2009). A monkey initiated a trial by touching the bar in the chair; 100 ms later a visual cue (13° on a side), which will be described below, was presented at the center of the monitor. After 500 ms, a red target (0.5° on a side) also appeared at the center of the monitor. After a variable interval of 500, 750, 1,000, 1,250, or 1,500 ms, the target turned green, indicating that the monkey could release the bar to earn a liquid reward. If the monkey responded within 200–1,000 ms, the target turned blue, indicating that the trial had been completed correctly. In correct trials, a reward of 1, 2, 4, or 8 drops of water (1 drop = ~0.1 mL) was delivered immediately after the blue signal. Each reward size was selected randomly with equal probability. The visual cue presented at the beginning of the trial indicated the number of drops for the reward. An inter-trial interval (ITI) of 1 s was enforced before the next trial began. If the monkey released the bar before the green target appeared or within 200 ms after the green target appeared or failed to respond within 1 s after the green target appeared, we regarded the trial as an “error trial”; all visual stimuli disappeared, the trial was terminated immediately, and, after the 1-s ITI, the trial was repeated. In this task, our behavioral measurement for the motivational value of outcome was the proportion of error trials. Since the monkeys were able to perform the task correctly in nearly every trial when the reward size was not assigned, an error trial is regarded as a trial in which the monkeys are not sufficiently motivated to correctly release the bar (Minamimoto et al. 2009). Before each testing session, the monkeys were subject to ~22 h of water restriction in their home cage. Each testing session continued for 120 min. Before the end of the session, the monkey received a sufficient volume of water (~300–500 mL) and stopped working spontaneously (usually at ~100 min). If the monkeys were allowed free access to water immediately after the session (i.e., after all behavioral experiments, see below), they still drank water, indicating that they were not completely satiated for water at the end of the session. During the behavioral testing session, blood samples were taken every 15 min (total of 8 samples; from 0 to 105 min). After all behavioral experiments, the monkeys were allowed free access to water. To measure the baseline osmolality level, we collected blood samples on 3 consecutive days at more than 2 months following the end of the experiment. In order to assess the natural fluctuations in blood osmolality, a blood sample was taken every 30 min (total of 5 samples; from 0 to 120 min) while a water-restricted monkey sat on a chair without any behavioral testing or access to water (3 sessions).

### Data analysis and model fitting

All data and statistical analyses were performed using the “*R*” statistical computing environment (R Development Core Team 2004). To assess the relationship between blood osmolality and cumulative reward, we performed multiple linear regression analysis. The osmolality level (*O*_SM_) was fitted by a liner regression model:

$$ O_{\text{SM}} = b_{0} + b_{\text{cum}} R_{\text{cum}} + b_{\text{sub}} S_{\text{ub}} , $$

where *R*_cum_ is the cumulative reward (mL), *S*_ub_ is the factor of subjects (0 and 1 for *Monkeys LP or GM*, respectively), *b*_cum_ and *b*_sub_ are the regression coefficient for cumulative reward and subject, respectively, and *b*_0_ is the intercept.

**Fig. 2:**
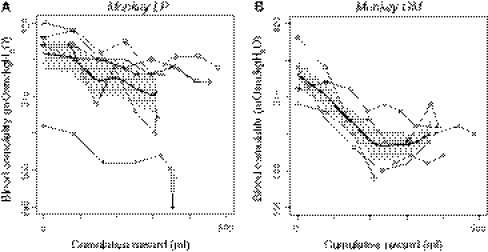
Changes in the osmolality level during reward accumulation. Changes in blood osmolality as a function of reward accumulation during the task performance in *monkey LP* (**a**) and *monkey* *GM* (**b**). A single session’s data, which were collected every 15 min, are plotted by distinctive symbols connected by *lines*. The *thick line* and shaded area represent the mean and SEM, respectively, for the change in osmolality with approximation (see text)

To assess the relationship between blood osmolality and task performance, we calculated the error rate for each drop-size condition within a 20-min time window around the blood sampling period (−12.5~7.5 min at each sample) (cf. Fig. 2). Each sample period contained 50 ± 23 and 54 ± 18 trials, in *Monkeys LP and GM*, respectively (mean ± SD).

We previously demonstrated that the error rate in the reward-size task has an inverse relationship with reward size: that is, *E* = *1/aR*, where *R* is the reward size, *a* is a constant parameter, and *E* is the error rate (%) of the monkeys in trials with reward size *R* (Minamimoto et al. 2009). Here, the motivational value of the expected outcome *R′* is inferred as being discounted as reward accumulation: $R^{\prime } = R \cdot F_{S} (S) = R\cdot\frac{{1 + e^{{ - (s - s_{0} )/\sigma }} }}{{1 + e^{{s_{0} /\sigma }} }}$, where *F*_*S*_(*S*) is the devaluation function of the normalized accumulated reward, *S*, *S*_0_ is the inflection point of the sigmoid, and *σ* quantifies the width of the sigmoid around *S*_0_. The normalized accumulated reward, *S*, which ranged from 0 (at the beginning of the session) to 1 (at the end of the session), was defined as the ratio between the amount of total reward delivered up to time *t*, *R*_cum_(*t*), and the total amount of reward, *R*_cum max_, delivered in the entire session: $S = \frac{{R_{\text{cum}} (t)}}{{R_{{{\text{cum }}\max }} }}$. Using the heuristic devaluation function, the inverse model was modified as: $E = \frac{1}{{aR \cdot F_{S} \left( S \right)}}$.

In this study, we tried to model the motivational value as being discounted as a function of the blood osmolality shift: $R^{\prime } = R \cdot F_{\text{OSM}} (S) = R \cdot \frac{{O_{\text{SM}} (S) - \rho }}{{O_{\max } - \rho }}$, where *F*_OSM_(*S*) is the osmolality devaluation function, which originated from the average blood osmolality change along with the reward consumption, *O*_SM_(*S*), *O*_max_ is the maximum value of *O*_SM_(*S*), and *ρ* is a free parameter corresponding to the threshold of the osmolality level, where the motivational value would be 0. The average blood osmolality change, *O*_SM_(*S*), was obtained individually as follows: Data for the changes in blood osmolality along with reward accumulation were linearly interpolated, and they were then averaged across sessions along with the cumulative reward from 0 to the smallest *R*_cum max_ among all sessions (thick lines in Fig. 2). It was then normalized by the smallest *R*_cum max_. Using the osmolality devaluation function, the inverse model was modified as: $E = \frac{1}{{aR \cdot F_{\text{OSM}} (S)}}$.

To examine the changes in the error rate along with water accumulation, the behavioral data from each session were divided into consecutive 9-quantiles with respect to the value of *S*; the error rate was evaluated in every 2 consecutive 9-quantiles, and thus, we obtained the error rate for 8 sub-sessions. These were then averaged across sessions for each sub-session. We fitted the models to these error data (4 reward size × 8 sub-sessions) with the least square minimization procedure described earlier (Minamimoto et al. 2009). The coefficient of determination (*R* ^*2*^) was reported as a measure of goodness of fit. Since these 2 models have a different number of free parameters, we used the Bayesian information criterion (BIC; BIC = − 2 × log-likelihood + *k*log*N*, where *k* is the number of free parameters and *N* is the number of data points) to compare the goodness of fit in each model.

## Results

We measured changes in blood osmolality by collecting 8 blood samples at 15-min intervals while the monkeys repeatedly consumed water rewards during the behavioral task (Fig. 1). The experiments continued until the monkeys stopped initiating new trials. We found that the blood osmolality level decreased as the cumulative reward increased through an entire daily session (Fig. 2). We noted that the profile of the declining osmolality level, according to water consumption, was fairly consistent across sessions within individuals, whereas it was different between monkeys (multiple liner regression analysis; significant negative effect of cumulative reward on osmolality, *p* < 0.001; main effect of subject, *p* < 10^−4^); osmolality declined gradually and linearly in *monkey LP*, but it declined steeply and then reached a plateau in *monkey GM* (Fig. 2, thick line). In a control experiment without rehydration (see “Methods”), osmolality did not change within 120 min (repeated measures ANOVA, *F*_(4, 8)_ = 0.4, *p* = 0.8), suggesting that the decrease in osmolality was not caused by natural fluctuations.

**Fig. 3:**
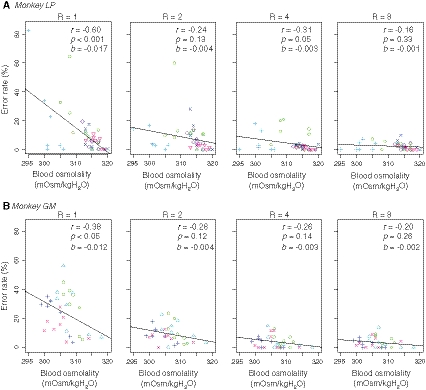
Relationship between task performance and osmolality level. The relationship between the error rate of the 1, 2, 4, and 8 drop trials and blood osmolality was plotted from left to right for *monkey LP* (**a**) and *monkey* *GM* (**b**). The error rates were calculated on the basis of a 20-min period around each blood sample. Each *symbol* represents the data from a single session

First, we sought to determine whether the hydration level accounts for the motivational value of water rewards and the motivation to earn the reward. To address this, we used the error rate in this task as a behavioral correlate of the motivational value because (1) an error trial in this task is regarded as a trial in which the monkeys are not sufficiently motivated to correctly release the bar and (2) the error rate is inversely related to the reward size, and its devaluation effect is inferred from reward accumulation (Minamimoto et al. 2009). We calculated the error rate for each reward-size trial performed at the time around blood sampling (see “Methods”). We found that there was a negative correlation between the error rate in the 1 drop condition and the blood osmolality level in each session [Fig. 3, left; *monkey LP*, *r* = −0.87~−0.59 (median −0.69); *monkey GM*, *r* = −0.92~−0.36 (median −0.77)]; the higher the osmolality level is (i.e., higher dehydration), the higher the motivation level. For the population data, there was a significant negative correlation between the error rate in the 1 drop condition and the blood osmolality level for each monkey (Fig. 3, left; *monkey LP*, *r* = −0.60, *p* < 0.001; *monkey GM*, *r* = −0.38, *p* < 0.05). This suggests that the value of 1 drop of water (and the monkey’s motivation to get it) depends on the hydration level. Although correlations between the error rates for the 2, 4, or 8 drop conditions and the osmolality level (*monkey LP*, *p* > 0.1; *monkey GM*, *p* > 0.05; see Fig. 3 in detail) were not statistically significant, the steepness of the regression slope became gentler as the reward size increased (i.e., the absolute value of *b* became small; repeated measures ANOVA, *F*_(3, 7)_ = 19.1, *p* < 0.05). These results suggest that the overall tendency of the error rate is subject to the expected reward size and blood osmolality.

**Fig. 4:**
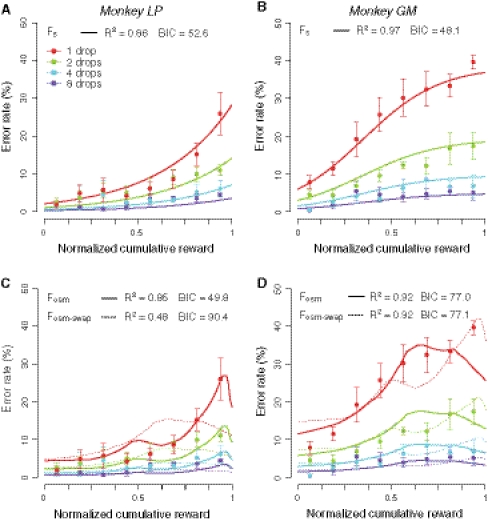
Explanation of task performance by a function of the cumulative reward and osmolality level. **a**–**d** The percentage of error trials (mean ± SE) for each reward size as a function of the normalized cumulative reward is shown for each monkey. **a**, **b** The *solid curves* are the best fit of Eq. 1 to the data. **c**, **d** The *solid* and *dotted curves* are the best fit of Eq. 2 to the data for their own osmolality shift function (*F*_OSM_) and from the other monkey (*F*_OSM-SWAP_), respectively

As we previously demonstrated (Minamimoto et al. 2009), the error rates in the reward-size task increased monotonically as the cumulative reward increased; this effect of the cumulative reward on error rates was consistent across reward size (Fig. 4a, b). This effect was modeled as the motivational value of outcome, *R′*, and was discounted as a function of the reward accumulation: *R′* = *R·F*_*S*_(*S*), where *R* is the reward size and *F*_*S*_(*S*) is the devaluation function of reward accumulation, *S* (Minamimoto et al. 2009). Thus, the inverse relationship between the error rate and reward size, *E* = *1/aR*, became:

1 $$ E = \frac{1}{{aR \cdot F_{S} (S)}} $$

The model explained the present data well (*monkey LP*, *R* ^*2*^ = 0.86; *monkey GM*, *R* ^*2*^ = 0.97, the best fit curves are in Fig. 4a, b, respectively), as seen in our previous study. Our aim here was to identify the mathematical form in which an index of physiological need for water (i.e., blood osmolality level) interacts with the expected reward size to determine the motivational value (i.e., error rate). We hypothesized that the value of water would be linearly discounted with decreasing osmolality and would become zero when the monkey is sated. To test this hypothesis, we formulated an osmolality-based devaluation function, *F*_OSM_(*S*), by using individually measured osmolality changes along with the cumulative reward (cf. Fig. 2), according to the following steps: (1) We extracted the average blood osmolality change, *O*_SM_(*S*), along with the reward consumption (thick curves in Fig. 2), where the actual reward consumption (0–*R*_cum max_) is normalized (0–1); and (2) in order to replace *F*_*S*_, we defined *F*_OSM_(*S*) as a normalized *O*_SM_(*S*), so that it ranges from 0 (at which value the monkey is not motivated for a water reward (i.e., satiated for water)) to 1 (at the maximum value of *O*_SM_, *O*_max_): $F_{OSM} (S) = \frac{{O_{\text{SM}} (S) - \rho }}{{O_{\max } - \rho }}$, where *ρ* is a free parameter corresponding to the threshold of the osmolality level at which the discounted value would be 0. This function gives us the ratio of water reward value discounted from the beginning of the task based on the linear transformation of the measured osmolality level. Note that *ρ* is not always necessarily equal to the osmolality level at which the monkeys stopped working spontaneously or at the end of the session, since the monkeys drank some water after the session if they were allowed free access. By replacing *F*_*S*_(*S*) with *F*_OSM_(*S*), we tried to explain the changing error rates according to the hydration level by the following equation:

2 $$ E = \frac{1}{{aR \cdot F_{\text{OSM}} (S)}} $$

The error rates were explained well by Eq. 2 for both monkeys (*monkey LP*, *R* ^*2*^ = 0.85; *monkey GM*, *R* ^*2*^ = 0.92, the best fit curves are in Fig. 4c, d, respectively). The best fit was given when *ρ* was 309 and 299 mOsm/kgH_2_O for *monkey LP* and *GM*, respectively. These values were in the range or higher than the normal osmolality level when the monkeys had free access to water (301 ± 4 mOsm/kgH_2_O for both monkeys), suggesting that the osmolality level at which the monkeys become satiated for water during the behavioral task varies among subjects. To compare the goodness of fit between the 2 models, Eqs. 1 and 2, we calculated the BIC. Eq. 2 was the best fit in *monkey LP* (BIC_Eq. 1_ = 52.6, BIC_Eq. 2_ = 49.8), whereas Eq. 1 was the best fit in *monkey GM* (BIC_Eq.1_ = 48.1, BIC_Eq.2_ = 77.0). Thus, both models explained the data comparably well.

Note that the course of increasing error rates was different between the monkeys: It increased gradually and then steeply in *monkey LP*, but it increased steeply and then gradually in *monkey GM* (Fig. 4c, d). This raises the possibility that individual differences in the change of the error rate are due to unique changes in the osmolality level (cf. Fig. 2a, b). To test this possibility, we tried to fit the model containing a swapped devaluation function, *F*_OSM-Swap_(*S*), where the normalized osmolality change was swapped between subjects. The swapped model never explained the data well; it did not fit the data of *monkey LP* (dotted curve in Fig. 4c, *R* ^*2*^ = 0.48), but it did fit the data of *monkey GM* to the same extent as the non-swapped model (dotted curve in Fig. 4d, *R* ^*2*^ = 0.92). This suggests that the monkeys’ motivation to obtain the water reward is adjusted on the basis of their own thirst level.

**Fig. 5:**
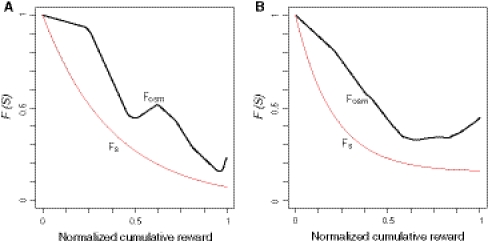
Comparison of the devaluation functions. Plotting the value of the devaluation functions with the best fit parameters against the normalized cumulative reward for *monkey LP* (**a**) and *monkey* *GM* (**b**). *F*_OSM_, devaluation function for their own osmolality; *F*_*S*_, devaluation function of the satiation level (sigmoid function)

Finally, we plotted two devaluation functions used for the fitting in Fig. 5 (the heuristic model, *F*_*S*_(*S*), and the osmolality model, *F*_OSM_(*S*)). Based on the heuristic or osmolality model, the water reward value at the beginning of the task was discounted as reward accumulation and becomes 20–50 % when the monkeys terminated the task.

## Discussion

Here, we found that as the total amount of water reward earned increased, the osmolality level, as one of the physiological indicators of the need for water, progressively decreased in an individual-dependent manner. There was a significant negative correlation between the error rate of the 1 drop trials and the osmolality level. Since the error rate of this task is a measure of motivational value (Minamimoto et al. 2009), this observation suggests that the motivational value of the water reward is computed in reference to the hydration level. Moreover, we found that the increase in the error rate with the cumulative reward received can be well modeled by the decrease in the osmolality level. Therefore, our results suggest that, under these conditions, the osmolality level is one of the major internal variables used in the computation of the motivational value of a water reward in the way that it is discounted as a reduction in the osmolality level.

### Measuring the relationship between the motivational value and hydration level

The blood osmolality level is one of the measurements of the hydration state in behaving monkeys (Yamada et al. 2010), and it has been used to reflect thirst or desire for fluids in numerous physiological studies (Bernardis and Bellinger 1996; Rolls and Rolls 1982). In this study, the osmolality level progressively decreased by 5–10 mOsm/kgH_2_O as a result of the intake of 300–400 mL water in ~105 min (Fig. 2). Previous studies reported a rapid decrease in blood osmolality; it decreased linearly at ~5 mOsm/kgH_2_O/60 min when monkeys received 150 mL water at a rate of 100 mL/60 min under controlled water access (Yamada et al. 2010), and it decreased by ~20 mOsm/kgH_2_O/20 min when monkeys drank water ad libitum (180 mL) after 24-h water deprivation (Wood et al. 1980). We found that the pattern for the change in the osmolality level together with water received was consistent across sessions, but in an individual-dependent manner. In one of our monkeys, the osmolality level stopped decreasing when the monkey received 200 mL water, and it then slightly increased (*monkey GM*; Fig. 2b). The individually unique change in the pattern might reflect the balance between the speed of water absorbance from the intestines and the speed of water excretion from the kidneys (Rolls and Rolls 1982).

We used the error rate as a behavioral correlate of the motivational value of reward in the reward-size task. Since the error trials of this task did not decrease, even if the monkeys were over trained and were rarely observed when the monkeys were well motivated (e.g., at the beginning of the session), they are mostly the consequence of reduced motivation and not, for example, of lessened motor ability or attention. Previously, we have shown that the error rate is inversely correlated with the expected reward size: *E* = *1/aR* with a devaluation function (cf. Eq. 1; Minamimoto et al. 2009). In this study, we found that there was a significant negative correlation between the error rate of the 1 drop trials and the osmolality level over all data in each subject (cf. Fig. 3, left), that is, the higher the osmolality level, the higher the reward value. This result suggests that the motivational value is discounted in parallel with the decrease in the blood osmolality level.

### Modeling motivational value by the hydration level

We confirmed our previous finding (Minamimoto et al. 2009) that the increase in the error rate of each reward size along with reward accumulation can be well explained by a devaluation function of satiation (i.e., how much the value of a certain amount of water is discounted as the normalized reward accumulation increases; cf. Eq. 1 and Fig. 4a, b). This devaluation function, *F*_*S*_, is a heuristic model; a sigmoid function was chosen since it explains many natural processes, and here, it satisfactorily explains the increase in the error rate with reward accumulation.

On the other hand, the osmolality devaluation function, *F*_*OSM*_, is modeled on the assumption that the reward value is discounted linearly as the hydration level increases. This assumption is supported by the observation that our data were explained by the osmolality devaluation function (cf. Eq. 2; Fig. 4c, d) as accurately as by the heuristic model (cf. Eq. 1; Fig. 4a, b).

Together with this assumption, we introduced a free parameter, *ρ*, in *F*_OSM_ as the upper threshold for osmolality at which the reward value would be discounted as 0. According to the best fit of Eq. 2, *ρ* was in the range or higher than the normal osmolality level when the monkey was assumed to have no physical need for water. A study in monkeys with an intravenous infusion of hypertonic saline found that the threshold for initiating drinking was ~7 mOsm/kgH_2_O above the normal level (Wood et al. 1982). The termination of drinking in thirsty pigs was correlated with a reduction in osmolality to the predeprivation level (Houpt et al. 1999). These results are consistent with our model in which the threshold for the drive for water (i.e., a positive value for the reward) is set above the normal osmolality level. Since the error rate and osmolality changed along with reward accumulation, one might claim that both are just simple parallel phenomena according to satiation for the reward. However, both factors changed in an individual-dependent manner, and the profile of the change in the osmolality level explained well the individually unique changes in the error rate. Although our individual-based analysis was limited (*n* = 2), the results support our model in which the motivational value was discounted linearly as the hydration level increased. It should be recognized that drinking behavior is also regulated by peripheral inputs (e.g., signals from sensory receptors in the digestive tract, e.g., those sensitive to gastric distension) (Maddison et al. 1980). Especially, these peripheral signals are suggested to be important for terminating a normal drinking bout, since the subject stops drinking long before the blood osmolality returns to normal (Wood et al. 1980); but see also (Houpt et al. 1999). In contrast to those voluntary rehydration conditions, the monkeys in our study slowly accumulated water rewards (e.g., ~500 mL in 100 min). Although our model did not consider those peripheral signals, the good fit of Eq. 2 (*R* ^*2*^ > 0.85) indicates that the osmolality level has a primary role in computing the motivational value of water rewards, at least under our experimental conditions.

In summary, our results suggest that the osmolality level is one of the primary internal variables used in the computation of the motivational water reward value. This extends our understanding of the role of the osmolality level in regulating drinking behavior from its initiation or termination to the adjustment of the motivation to engage. In our improved model, the motivational value of water rewards is discounted as a reduction in the osmolality level to the threshold for the drive for water.

### Possible neural circuit for the evaluation of water rewards on the basis of the hydration level

Our results suggest that the osmolality level is one of the main internal variables for the calculation of the motivational value; besides, osmolality seems to be one of the physiological variables that correlate with the drive for drinking. Spontaneous drinking is elicited after the blood osmolality level is increased by ~10 mOsm/kgH_2_O following an intravenous infusion of hypertonic saline (Anderson and Houpt 1990; Egan et al. 2003). This hypertonic-induced drinking is prevented by lesions of the organum vasculosum laminae terminalis (OVLT), which is one of the brain’s circumventricular organs that lies outside of the blood–brain barrier (Thrasher and Keil 1987; Thrasher et al. 1982). Discharges of a subset of neurons in the OVLT increase as a function of fluid osmolality (Ciura and Bourque 2006; Sayer et al. 1984). Functional MRI studies have also shown that the anterior region of the third ventricle becomes activated during the onset of extracellular fluid hypertonicity in humans (Egan et al. 2003). Thus, neurons in the OVLT seem to serve as the primary osmoreceptors that transduce the osmolality level into neuronal signals.

In rats, there are direct projections from the preoptic region of the brain containing the lamina terminalis to the paraventricular (PV) and mediodorsal (MD) thalamic nuclei (Chiba and Murata 1985; Ray et al. 1992; Van der Werf et al. 2002), which have been implicated in osmotic signaling (Gonzalez-Lima et al. 1993; Hall 1989; Hollis et al. 2008). Especially, the MD is also suggested to have a role in reinforcer devaluation (Izquierdo and Murray 2010). The orbitofrontal cortex (OFC), with direct connections to the MD, is also suggested to have a role in reinforcer devaluation (Izquierdo et al. 2004) as well as representing the predictive reward value (Gottfried et al. 2003; Padoa-Schioppa and Assad 2006). Ablation of the bilateral OFC disrupts normal reward value estimation in the reward-size task, but it does not affect the internal drive state (Simmons et al. 2010), suggesting that the OFC is not the only brain region involved in the calculation of the motivational reward value on the basis of external and internal variables. Two other cortical areas, the ventromedial prefrontal cortex (VMPFC) and the insular cortex (INS), with direct connections to the two thalamic nuclei, are also suggested to have a role in the sensation of thirst and the drive to drink. For example, imaging studies in humans have shown that changes in the activity of the VMPFC and INS correlate with the progressive intensification of thirst and its satiation upon drinking (Denton et al. 1999). Electrical stimulation of parts of the VMPFC has been found to elicit drinking within seconds of stimulus onset in awake monkeys (Robinson and Mishkin 1968). A recent electrophysiological study demonstrated that the neuronal activity of the VMPFC during a reward-size task is affected by satiation for a water reward (Bouret and Richmond 2010).

In summary, the PV and MD thalamic nuclei, OFC, VMPFC, and INS form the possible neural basis for the motivational value of water rewards depending on the hydration level. A future study will be required to identify the neural network involved in computing the motivational value of water rewards from the hydration level and external variables. Our model-based approach, combining a reward-size task and the measurement of osmolality, is a promising strategy to identify this network.
